# The Maturation of Interference Suppression and Response Inhibition: ERP Analysis of a Cued Go/Nogo Task

**DOI:** 10.1371/journal.pone.0165697

**Published:** 2016-11-04

**Authors:** Laura Vuillier, Donna Bryce, Denes Szücs, David Whitebread

**Affiliations:** 1 Department of Psychology, Bournemouth University, Bournemouth, United Kingdom; 2 Department of Psychology, University of Tübingen, Tübingen, Germany; 3 Department of Psychology, University of Cambridge, Cambridge, United Kingdom; 4 Faculty of Education, University of Cambridge, Cambridge, United Kingdom; Tsinghua University, CHINA

## Abstract

Inhibitory control is a core function that allows us to resist interference from our surroundings and to stop an ongoing action. To date, it is not clear whether inhibitory control is a single process or whether it is composed of different processes. Further, whether these processes are separate or clustered in childhood is under debate. In this study, we investigated the existence and development of two hypothesized component processes of inhibitory control–interference suppression and response inhibition–using a single task and event related potential components. Twenty 8-year-old children and seventeen adults performed a spatially cued Go/Nogo task while their brain activity was recorded using electroencephalography. Mean N2 amplitudes confirmed the expected pattern for response inhibition with both the children and the adults showing more negative N2 for Nogo vs. Go trials. The interference suppression N2 effect was only present in adults and appeared as a more negative N2 in response to Go trials with a congruent cue than Go trials with an incongruent cue. Contrary to previous findings, there was no evidence that the interference suppression N2 effect was later occurring than the response inhibition N2 effect. Overall, response inhibition was present in both the children and the adults whereas interference suppression was only present in the adults. These results provide evidence of distinct maturational processes for both component processes of inhibitory control, with interference suppression probably continuing to develop into late childhood.

## Introduction

Inhibitory control, the ability to resist interference or inhibit ongoing actions, is considered an important executive function that allows people to maintain and achieve a goal in novel problem solving situations (e.g. [1]). However, recent research has indicated that inhibitory control may actually be composed of two component processes, often referred to as interference suppression and response inhibition (e.g. [2]). These component processes need to be thoroughly investigated, and reliable measures of them established, since there is evidence that they develop differently [2] and contribute differently to developmental disorders such as ADHD (e.g. [3,4]). To date, much of the research into interference suppression and response inhibition has used different tasks to measure each one, and/or been based on only behavioral data (but see [2,5,6] for promising neurophysiological data). Thus, in order to contribute to the existing knowledge in this field, in the current study we measured electroencephalography (EEG) during one task that placed demands on both component processes of inhibitory control (a spatially cued Go/Nogo task). Additionally, we examined the suitability of such a task for young children, and whether the neural markers of each component process were sensitive to developmental change, by collecting data from both 8-year-olds and adults.

Over the last decade, various terms have been used to define the two proposed component processes of inhibitory control, such as interference suppression and response inhibition [2], stimulus interference control and response interference control [7], selective and nonselective inhibition [8], and change and stop responses [5]. Here, we use the term *interference suppression* to refer to resisting interference from irrelevant or misleading information, and *response inhibition* to refer to stopping a prepotent response. Interference suppression is thought to be predominantly required in Stroop [9] and Flanker [10] tasks, in which the irrelevant information (the written word, or flanker stimuli, respectively) must be suppressed in order to respond appropriately in all trials (by naming the ink color, or responding to the central target stimulus, respectively). Importantly, in incongruent trials of these tasks the irrelevant and relevant information conflict with one another, thus requiring a ‘change’ of response preparation. Response inhibition, on the other hand, is typically required most in Go/Nogo (e.g. [11]) or Stop Signal [12] tasks, in which the response must be stopped (withheld completely, or halted in response to a signal, respectively). Performance on these tasks provides some evidence that the two processes of inhibitory control are dissociable. For instance, Huizinga et al. [13] found that performance on Stroop, Stop Signal and Flanker tasks were not consistently correlated, and sometimes even negatively correlated, suggesting they do not tap one common inhibitory control skill. There is also evidence that these two processes, although distinct, might be related (e.g [14,15]) and some recent work even suggested that a super-ordinate cognitive control network may be involved in all executive functions [16,17], suggesting a possible general mechanism common to both processes. However, while these classic inhibitory control tasks are considered to predominantly make demands on one or the other process, it is likely that both are used to some extent in all inhibitory control tasks and that behavioral measures, such as reaction times, represent an amalgamation of these skills.

In contrast, electrophysiological studies have examined the brain activity related to inhibitory control in the time preceding a correct response in such classic inhibitory control tasks. The N2 component, a negative deflection around 150–400 ms at frontocentral electrode sites, is considered a marker of inhibitory control since it often differs between conditions in these classic inhibitory control tasks. With respect to interference suppression, the direction of this effect is unclear, with some Stroop studies finding a larger (more negative) N2 on congruent compared to incongruent trials [18,19] and others finding the opposite effect [20,21]. The N2 is more consistently found to be enhanced for incongruent than congruent trials in the Flanker task (e.g. [22,23]); however, more recent findings on the effect of increasing the frequency of incongruent trials on the N2 have raised questions about what the component really reflects in this task [24]. The effect of response inhibition on the N2 is more reliable–a greater (more negative) N2 is typically observed in Nogo compared to Go trials in a Go/Nogo task [25–27].

It is clearly problematic to draw conclusions regarding the differences between interference suppression and response inhibition by comparing behavioral performance or neural activity across different single tasks. That is, many other task demands that are unrelated to inhibitory control could vary across tasks, and methodology and the approach to analysis undoubtedly vary across different studies. To address this, some EEG studies have attempted to determine whether interference suppression and response inhibition are truly functionally different skills by adapting classic tasks to create so-called ‘hybrid tasks’ that make demands on both processes [5,6]. Krämer et al. [5] used an adapted version of a Stop Signal task in which participants had to either stop their response (requiring response inhibition) or change their response (requiring interference suppression). While they found a response inhibition related effect on the frontal N2 (maximal at 240 ms), the N2 did not change as a function of interference suppression demands. In contrast, Brydges et al. [6] observed an effect of both types of inhibitory control on the N2 using a hybrid Go/Nogo flanker task. Their results indicated that the N2 effect related to interference suppression (a comparison of congruent Go and incongruent Go conditions) was maximal at central electrodes, and occurred relatively late (between 356 and 480 ms), whereas the N2 effect related to response inhibition (a comparison of congruent Go and congruent Nogo conditions) was maximal at frontal electrodes, and occurred relatively early (between 256 and 300 ms). In summary, it seems that an enhanced frontal N2 is fairly reliably observed when participants must use their response inhibition, but the findings regarding interference suppression are less clear-cut. We aimed to contribute to these emerging findings in the current study, also using an adapted Go/Nogo task.

As previously mentioned, our secondary aim was to examine developmental differences in interference suppression and response inhibition. The degree to which these are two distinct processes can be informed by establishing whether they develop in different regions and / or at different rates. An early fMRI study that compared the brain activity related to each process in 8–12-year-old children and adults found evidence that the groups used different brain areas for suppressing interference and inhibiting responses [2]. However, the developmental changes were more distinct for response inhibition than for interference suppression; when response inhibition was required, children showed activity in a subset of the areas that adults did, and when interference suppression was required both groups engaged the prefrontal cortex, but opposite hemispheres. Similarly, when Brydges et al. [28] administered their hybrid Go/Nogo flanker task to both adults and 8–11-year-old children, they found significant developmental differences in the neural activity related to response inhibition, but the results concerning interference suppression were less clear. That is, the N2 effect associated with response inhibition was maximal at central electrodes in children, becoming more frontal by adulthood, and also occurred earlier in adults than in children. These differences are consistent with the observation that with age, gray matter reduces and white matter volume increases in regions involved with inhibitory control and that the areas recruited shift from posterior to anterior [28,29]. However, since there was no difference between the N2 elicited in congruent Go and incongruent Go conditions in children, it was not possible to draw conclusions about the development of interference suppression. This latter finding could have been due to three methodological features of this study, namely the small sample size of 13 in each group, the large age range of the children, and the young age of the adult group (they were 18 years, and it has been suggested that inhibitory control is not fully developed until at least 21 years [30]). We propose that our study, with a larger sample size, smaller age range of children, and older adults can complement these findings.

Another EEG phenomenon, the Lateralised Readiness Potential (LRP), has also been used to measure inhibitory control in a range of classic tasks (e.g. Stroop: [31]; Stop Signal: [32]; Flanker: [33]). The LRP is a measure of response preparation that is observable before a latent behavioral response. Thus, using the LRP it is possible to directly observe the time-course of inhibitory control being asserted at the central activation stage. This phenomenon seems to be sensitive to the influence of irrelevant information on motor preparation over time (i.e. the temporal dynamics of interference suppression), since in the incongruent condition of a Stroop-like task an incorrect response preparation that is then overcome to give a correct response has been observed [34,35]. While this effect has been observed in participants as young as 5 years [35], incorrect response preparation in the incongruent condition is not always observed (e.g. in children with ADHD, Kòbor et al. [36]; and in adults completing a numerical Stroop task, [31,37]). Nevertheless, in the current study the LRP was used to examine the effect of a spatial cue on participants’ motor preparation and as an additional measure of the temporal properties of interference suppression.

In the current study, we administered a new spatially cued Go/Nogo task that made demands on the two hypothesized component processes of inhibitory control to 8-year-old children and adults, while recording EEG. The target stimuli (which could be Go or Nogo) were presented above or below a fixation point, and participants had to respond as fast as possible to a Go stimulus (using the corresponding response key, ‘above’ or ‘below’) and withhold their response completely for a Nogo stimulus. By adding a cue (which was 65% congruent) indicating the location where the target stimulus would appear, we aimed to create a condition that is similar to the incongruent condition of a Stroop task. That is, when the cue indicates that the stimulus will appear above the fixation point, the participant will prepare an ‘above’ response. However, if the stimulus then appears below the fixation point (i.e. if the cue and target are incongruent), the participant must suppress their initial incorrect motor preparation, in order to give a response with the other hand. This is analogous to, in the classic color-word Stroop task, the participant suppressing the incorrect response based on the word, in order to give an alternative response based on the ink color. Thus, comparing behavioral data and the neural activity associated with congruent and incongruent cues to Go target stimuli informs us about interference suppression, while comparing the neural activity associated with congruent Go and congruent Nogo trials informs us about response inhibition.

Based on previous findings using a Stroop-like task [35,38] the LRP was analyzed in order to track response preparation in the pre-target (response preparation) interval. If participants were preparing responses based on the cue, we expected this to be reflected in the LRP. Importantly, we expected to see a response preparation in the direction of the cue for all trials in the response preparation interval, followed by a response preparation in the direction of the target for Go trials only in the response execution interval. Further, the N2 in response to the presentation of the target stimulus was examined. Based on the findings of Brydges and colleagues [6,28], we expected the N2 to be enhanced (more negative) in the congruent Go as compared to incongruent Go condition (interference suppression N2 effect) and that this effect would be maximal relatively late at central locations (at least in the adult group). Also based on the Brydges et al. findings [6], we expected the N2 to be greater (more negative) in the congruent Nogo than in the congruent Go condition (response inhibition N2 effect) and that this effect would be maximal relatively early at frontal locations (again, at least in the adult group).

Given the limited literature on the development of these component processes, we did not have strong hypotheses regarding the comparison of child and adult data. However, these data could provide novel information about the development of interference suppression and response inhibition, and suitable measures of these processes.

## Methods

### Participants

Originally, twenty children (5 male) from Cambridgeshire and seventeen adults (5 male) participated in the experiment. Before running any analyses, the data from two children (1 male) and one female adult were rejected because of EEG artefacts. The mean age of the remaining eighteen children was 8 years and 5 months (range = 8 years 1 month to 9 years 5 months, SD = 0.42). The mean age of the remaining sixteen adults was 26 years and 2 months (range = 23 years 1 month to 29 years 7 months, SD = 2.21). All analysis occurred after artefact rejection was complete in all participants. All the participants were right-handed. Adults were graduate and undergraduate students and staff at the University of Cambridge. Children received a T-shirt as a token of gratitude for participation. Adults received £16 for their participation. This study received the approval of the University of Cambridge ethics committee. Written informed consent was obtained from adult participants and from a parent/guardian for child participants.

### Stimuli and Procedure

**Fig 1:**
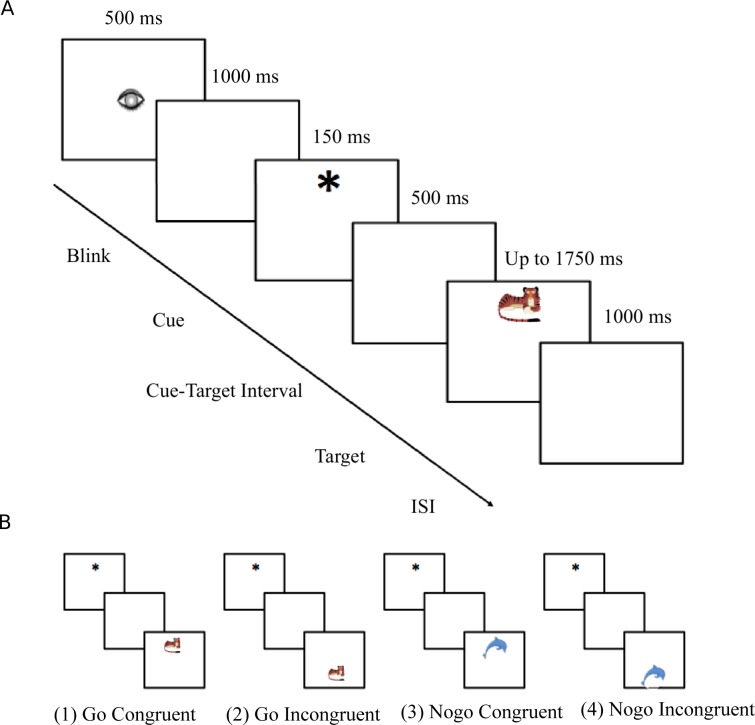
Task and experimental stimuli. (A) The 6 screen pictures represent the different stages of the task. (B) Stimuli: examples of the four possible conditions. In this example, the participants were asked to feed the orange animals (i.e, orange animals were Go trials and blue animals were Nogo trials). (1) Go Congruent (GC); (2) Go Incongruent (GI); (3) Nogo Congruent (NGC) and (4) Nogo Incongruent (NGI). The cue was presented in the top or in the bottom half of the screen. Four different animals for each color were used.

Stimuli were colored pictures of animals presented on a 17-in computer screen. The participants were seated 100 cm away from the screen. Only one animal was presented at a time, in the top or the bottom half of the screen. Stimuli were orange animals “from the land” (lion, leopard, tiger and puma) and blue animals “from the sea” (dolphin, whale, orca, fish). Half of the participants were asked to feed the orange animals and the other half were asked to feed the blue animals by pressing on a button when they saw a ‘Go’ animal (i.e. the one they had to feed) and to withhold their response for a ‘Nogo’ animal. 80% of the trials were Go, 20% were Nogo. A cue (small asterisk) preceded the presentation of the animal and was presented for 150 ms in the top or the bottom half of the screen. This cue predicted the location of the animal 65% of the time, although no such information was given to the participants. The trial was called congruent if the cue and the animal appeared at the same location; and incongruent if the cue and the animal appeared in different halves of the screen (see Fig 1). The cue and the targets were presented at a visual angle of 1° and 2° respectively. The inter-trial interval was 1000 ms. The animals were equally and randomly presented in the top or the bottom half of the screen. Participants gave behavioral responses by pressing a button on a game pad with the left or right thumb. For half of the participants, the top button was on the left side, for the other half it was on the right side. The cue and the stimulus were presented to the top or to the bottom of the screen to ensure that both visual fields (left and right) were equally stimulated, thus avoiding any confounding brain activity in the LRP analyses.

Each trial consisted of a fixation sign (drawing of an eye) shown for 500 ms followed by a 1000 ms blank period. Then, the cue was presented for 150 ms, and the stimulus was presented after a 500 ms blank-screen and for a maximum of 1750 ms. The stimulus disappeared when the participant gave a response (or stayed on the screen for the maximum period for Nogo trials). The offset of the stimulus was followed by a 1000 ms blank screen. Participants were advised to blink only when they saw the drawing of an eye. The experiment took on average 1.5 h to complete. The children completed five blocks of 90 stimuli (altogether 248 Go Congruent trials, 112 Go Incongruent trials, 45 NoGo Congruent trials and 45 Nogo Incongruent trials) and the adults completed 10 blocks. The experiment was preceded by 20 practice trials. Stimuli were presented by the Presentation program of the Neurobehavioral Systems (San Fransisco, CA, USA). Data were recorded in an acoustically and electrically shielded testing booth.

### ERP recording and pre-processing

EEG was recorded by an Electrical Geodesics system with a 64-channel Geodesic Sensor Net for children and a 128-channel Geodesic Sensor Net for adults. The sampling rate was 500 Hz. An on-line lowpass filter of 70 Hz was used. The data were band-pass filtered between 0.03 and 30 Hz off-line and were recomputed to average reference. Epochs extended from −850 to +850 ms relative to stimulus presentation. Data were baseline corrected by a baseline of −200 to 0 ms relative to stimulus presentation (for N2 analyses), or −200 to 0 relative to the cue presentation (for LRP analyses). Epochs containing ocular artefacts (monitored at electrodes below, above, and next to the eyes) and epochs containing voltage deviations exceeding ±200 μV relative to baseline at any of the recording electrodes were rejected. The maximal allowed voltage step was 50 μV/ms. Participants with less than 60% of all trials accepted after artefact rejection were excluded from the sample (two children and one adult). On average, 76% of trials were kept in children and 89% in adults.

Averaged ERPs were computed for each participant in the four different conditions: (1) Go Congruent trials (GC); (2) Go Incongruent trials (GI); (3) NoGo Congruent trials (NGC); and (4) NoGo Incongruent trials (NGI). Only correct response (Go) or correct no response trials (Nogo) were included in the averaging procedure. After artefact rejection and selection of correct trials, an average of 287 trials were kept per child participant (172 GC, 79 GI, 32 NGC and 4 NGI) and 747 per adult participant (436 GC, 193 GI, 78 NGC and 40 NGI).

### Data analysis

#### Behavioral analysis

Mean accuracy in all conditions was analyzed in an ANOVA with the between-subjects factor Group (children, adults) and the within-subjects factor Condition (GC, GI, NGC, NGI). Mean reaction time (RT) to correctly responded Go trials was analyzed in a mixed design ANOVA with a between-subjects factor Group (children, adults) and a within-subjects factor Condition (GC, GI). The Greenhouse–Geisser correction was used to adjust p-values where appropriate, and partial eta-squared effect sizes are provided.

#### Pre-stimulus LRP analysis

**Fig 2:**
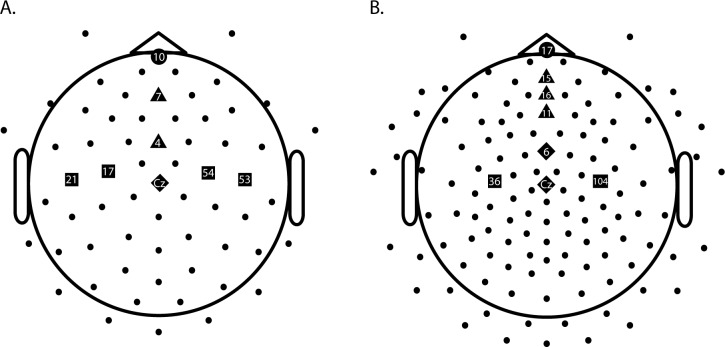
**Electrodes used for EEG analyses for children (A) and adults (B).** Note: squares depict electrodes used for LRP calculation, circles those used for the frontal N2, triangles those used for the fronto-central N2, and diamonds those used for the central N2.

The LRP for all four conditions was computed as proposed by Coles [39]:

(ER−EL)lefthandresponse+(EL−ER)righthandresponse/2

where ER represents the activity from an electrode situated over the right motor cortex (usually C4 in the 10–20 electrode system), and EL represents the activity from an electrode situated over the left motor cortex (usually C3). In children, EL and ER were calculated as an average of two electrodes that were the closest to the C3 and C4 position (electrodes 17 and 21 for C3; 53 and 54 for C4). For the adults, electrode 36 was used for C3 and 104 for C4. Electrode positions are depicted as squares in Fig 2. These electrode choices were consistent with the study of Bryce et al. (2011), and the LRP had the expected morphology and timing that were previously observed in our group [34,35,38,40]. In these studies, a negative LRP indicates correct response preparation, and a positive LRP indicates incorrect response preparation.

In the current study, the LRP was computed in such a way that a preparation in the direction indicated by the cue was considered a correct response preparation. Hence, if there was pre-stimulus response preparation in response to the cue then the LRP should deviate into the negative direction in all conditions after cue presentation and before stimulus presentation. We examined whether the LRP significantly deviated from baseline (zero) at all in the response preparation interval. As in previous studies [34,35,37,40–42], the deviation of the LRP from the baseline was tested by point-by-point two-tailed one-sample t-tests against zero (*p* < .05). Deviations from zero were considered significant if they persisted for at least 20 consecutive time points. We also measured the LRP in the response execution interval (after the stimulus presentation) in the 0–800ms interval following the same procedures.

#### Post-stimulus N2 analysis

Consistent with Brydges et al. [28], analysis of the N2 component was based on activity from frontal (Fz), fronto-central (FCz) and central (Cz) locations on the midline of the scalp. For the children, frontal activity was measured at electrode 7, fronto-central activity was measured at electrode 4, and central activity was measured at electrode Cz. For the adults, frontal activity was measured at electrode 11, fronto-central activity was measured at electrode 6, and central activity was measured at electrode Cz. See Fig 2 for electrode positions.

The mean N2 amplitudes and peak latencies were calculated within windows that were determined on the basis of inspection of the grand average waveforms. In line with the literature showing that the N2 peaks later in children compared to adults [28], in children this window was 300–400 ms, and in adults this window was 200–300 ms.

Mean N2 amplitudes and peak latencies were both analyzed in mixed design ANOVAs with the between-subjects factor of Group (children, adults) and the within-subjects factors of Condition (GC, GI, NGC) and Location (frontal, fronto-central, central). Significant effects within each age group were further examined by separate Condition (3) x Location (3) repeated measures ANOVAs. The Greenhouse–Geisser correction was used to adjust p-values where appropriate, and partial eta-squared effect sizes are provided. In the case of significant main effects, Tukey post hoc tests were conducted.

In order to be consistent with the studies of Brydges et al. [6,28] and to better evaluate whether interference suppression and response inhibition are separable processes, we additionally computed and analyzed N2 difference waves for each of the component processes of inhibitory control. That is, for each participant, mean EEG activity during GC trials was subtracted from mean EEG activity during GI trials in order to generate the interference suppression effect difference wave. Likewise, mean EEG activity during GC trials was subtracted from mean EEG activity during NGC trials in order to generate the response inhibition effect difference wave. Mean amplitudes and peak latencies within the same time windows and locations as above were computed and analyzed in ANOVAs, with the between-subjects factor of Group (2) and the within-subjects factor of Location (3).

For ease of reading, only significant effects are reported throughout the Results. The datasets are available in the supporting information with the S1 File for ERP mean amplitudes and latencies and the S2 File for reaction time and accuracy.

## Results

### Behavioral results

The behavioral results are reported in Table 1. Overall, children responded less accurately than adults, *F*(1, 32) = 7.97, *p* = .008, *η*_p_^2^ = .20, but no other effects on accuracy were significant. Children also responded more slowly to Go trials than adults, *F*(1, 32) = 44.09, *p* < .001, *η*_p_^2^ = .58, and a main effect of Condition indicated that RTs were shorter to GC trials than GI trials, *F*(1, 32) = 30.26, *p* < .001, *η*_p_^2^ = .49. This main effect of Condition was also significant in each separate group ANOVA (children: *F*(1, 17) = 22.30, *p* < .001, *η*_p_^2^ = .57; adults: *F*(1, 15) = 10.18, *p* = .006, *η*_p_^2^ = .40).

**Table 1:** Mean (standard error) accuracy (% correct) and reaction time (ms) in children and adults, for each condition.

	Accuracy (%)				Reaction time (ms)	
	GC	GI	NGC	NGI	GC	GI
Children	94.2 (2.1)	95.2 (1.8)	92.4 (2.5)	94.5 (2.7)	585 (16)	618 (15)
Adults	99.0 (0.5)	98.9 (0.4)	99.5 (0.2)	99.4 (0.3)	423 (19)	452 (23)

Note: GC = Go congruent; GI = Go incongruent; NGC = Nogo congruent; NGI = Nogo incongruent.

### ERP results

#### LRP

**Fig 3:**
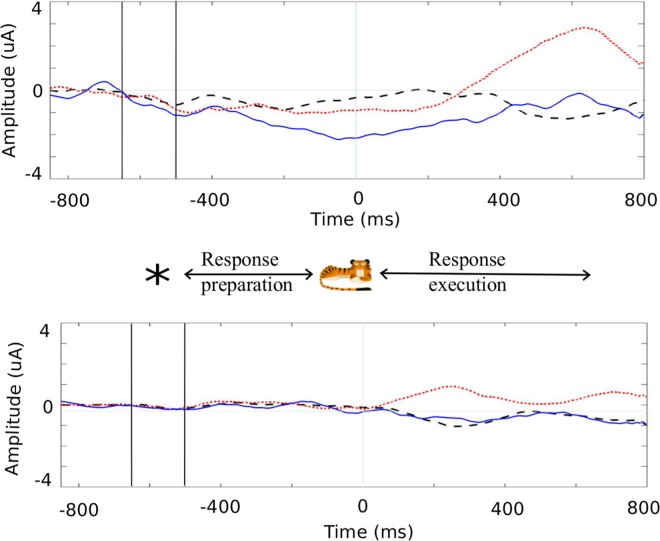
**Cue-locked LRP for Go Congruent (GC) (black dashed line), Go Incongruent (GI) (red dotted line) and Nogo Congruent (NGC) (blue solid line) conditions in children (A) and adults (B).** Note that a negative deflection in the response preparation interval (before 0 ms) reflects a preparation in the direction of the cue; in the response execution interval (after 0 ms) continued negative deflection in Go Congruent trials and positive deflection in Go Incongruent trials reflect correct response preparation.

During the pre-stimulus response preparation interval (before time 0 ms), the LRP deviated negatively from baseline in all three conditions for the children (GC: -534 to -478 ms and -310 to -120 ms; GI: -528 to -368 ms and -106 to 0 ms; NGC: -600 to -546 ms and -242 to 0 ms). In the adults, the LRP did not significantly differ from zero. As a negative deviation means that there was a pre-stimulus response preparation in the direction of the cue, the LRP findings indicate that the cue influenced the motor stage in the children, but did not influence the motor stage of the adults. As can be seen in Fig 3, both the children and the adults showed correct motor activation in the response execution interval (after time 0 ms) for all Go trials (after the presentation of the stimulus, corresponding to when they had to respond to the Go target). For NGC, the LRP in the response execution interval was not significantly different from zero in either group, reflecting no motor activation in Nogo trials (as expected).

#### N2 mean amplitude

**Fig 4:**
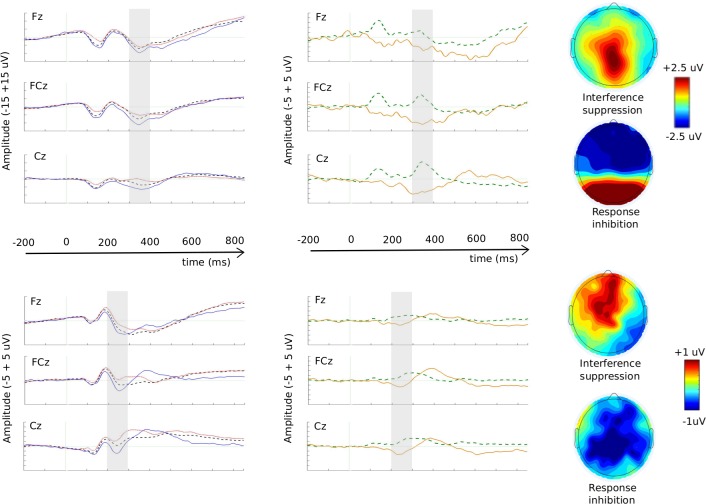
The N2 component. Stimulus-locked grand average ERP waveforms in response to GC (black dashed line), GI (red dotted line) and NGC (blue solid line) at electrodes Fz, FCz and Cz, for children (A) and adults (D). Grand-averaged difference waveforms computed as GI–GC (green dashed line; *interference suppression N2 effect*) and NGC–GC (yellow solid line; *response inhibition N2 effect*) at electrodes Fz, FCz and Cz, in children (B) and adults (E). Note: The window used for N2 analysis in each group is marked by the grey area (300-400ms for the children and 200-300ms for the adults). The topographies represent the difference within each window of analysis for interference suppression (up) and response inhibition (down) in children (C) and adults (F). Please also note that the scale for the ERP waveforms is different between the children and adults’ data; being -15 +15 μV for the former (A) and -5 +5 μV for the latter (D)

The N2 component can be seen in Fig 4. Overall, children had more negative mean N2 amplitudes than adults, *F*(1, 32) = 47.54, *p* < .001, *η*_**p**_^2^ = .60. The N2 was more negative for the NGC condition than the two other conditions, *F*(2, 64) = 17.52, *p* < .001, *η*_**p**_^2^ = .35 (post hoc tests *p*s < .01), and more negative at both frontal and fronto-central locations than at the central location, *F*(2, 64) = 15.35, *p* < .001, *η*_**p**_^2^ = .32 (post hoc tests, *p*s < .001). Both of these main effects interacted with Group (Condition x Group: *F*(2, 64) = 9.70, *p* < .001, *η*_**p**_^2^ = .18; Location x Group: *F*(2, 64) = 10.13, *p* = .001, *η*_**p**_^2^ = .24), indicating that these amplitude difference effects were greater in children than in adults.

The repeated measures ANOVA on the child data showed essentially the same result pattern as the omnibus ANOVA. The N2 was greater (more negative) in the NGC condition than the GC (*p* = .005) and GI (*p* < .001) conditions, *F*(2, 34) = 14.46, *p* < .001, *η*_p_^2^ = .46, and at the frontal (*p* < .001) and fronto-central locations (*p* < .001) than at the central location, *F*(2, 34) = 13.55, *p* < .001, *η*_p_^2^ = .44.

While the Condition main effect was also significant in the ANOVA on the adult data, *F*(2, 30) = 12.81, *p* < .001, *η*_p_^2^ = .46, the post hoc tests revealed a different pattern than in the child data–all three conditions were significantly different from each other (NGC < GC < GI, *p*s < .05). Mean N2 amplitude was also affected by location, *F*(2, 30) = 10.35, *p* = .003, *η*_p_^2^ = .41, and as in the child data, the N2 was greater (more negative) at fronto-central and frontal locations than at the central location (*p* = .035 and *p* < .001, respectively).

Regarding the interference suppression related N2, the difference between GC and GI conditions did not reach significance in the child group–contrary to our hypothesis–but the adult group did show the expected effect with a larger N2 in the GC than in the GI condition. Consistent with our hypothesis regarding the response inhibition related N2, we observed a greater N2 in response to NGC than GC in each age group.

#### N2 peak latencies

Unsurprisingly, given that we measured peak latencies within different windows in each age group, the N2 peaked later in children (353 ms) than in adults (257 ms), *F*(1, 32) = 426.66, *p* < .001, *η*_p_^2^ = .93. When a longer analyses window was used (200 – 400ms time window for both groups) the N2 still peaked later in children than adults, *F*(1, 32) = 43.57, *p* < .001, *η*_p_^2^ = .58.

Further, the N2 was affected by Location, *F*(2, 64) = 5.89, *p* = .008, *η*_p_^2^ = .16, and reached its peak later at the frontal than at the central location (*p* = .014). A significant Location x Group interaction, *F*(2, 64) = 14.42, *p* < .001, *η*_p_^2^ = .31, indicated that the effect of location on N2 peak latency was different in each age group.

When conducted only using the child data, the repeated measures ANOVA on N2 peak latency showed no significant effects. In contrast, in the adult group the peak of the N2 component was affected by electrode location, *F*(2, 30) = 14.54, *p* < .001, *η*_p_^2^ = .49. Specifically, it peaked first at the central location (243 ms), followed by the fronto-central location (257 ms), and then the frontal location (271 ms; all post hoc comparisons *p* < .05).

The peak latency results offered no support for our hypothesis that the interference suppression N2 effect would occur later than the response inhibition N2 effect, as there were no significant effects of Condition on peak latency.

#### N2 difference waves: Interference suppression

Since we did not observe a significant difference between the N2 in response to GI and GC conditions in the children, the GI–GC difference wave (the interference suppression N2 effect) in the children was not analyzed further. In adults, while the mean amplitude of the GI–GC difference wave was not affected by Location, the peak latency was, F(2, 30) = 4.63, p = .018, η_**p**_^2^ = .24. Post hoc tests showed that the effect peaked later at the fronto-central than at the frontal location (p = .007).

#### N2 difference waves: Response inhibition

The NGC–GC difference wave (response inhibition N2 effect) had a greater amplitude in children than adults, F(1, 32) = 11.81, p = .002, η_**p**_^2^ = .27. However, in contrast to the interference suppression effect, location had a significant effect on the mean amplitude in the adult group, F(2, 30) = 4.03, p = .045, η_**p**_^2^ = .21. Post hoc tests indicated that the effect was greater at the central location than the frontal location (p = .013). This was contrary to our hypothesis that the response inhibition effect would be maximal at frontal sites. The peak latency of the NGC–GC difference wave was later in children than adults, F(1, 32) = 162.55, p < .001, η_**p**_^2^ = .84. Otherwise, there were no significant effects on the peak latencies of the response inhibition N2 effect. Further, there was no evidence that the response inhibition effect peaked earlier than the interference suppression effect (mean peak latencies of 309 vs. 304 ms, respectively).

## Discussion

In the present study we aimed firstly to investigate whether interference suppression and response inhibition are separable component processes of inhibitory control using a hybrid cued Go/Nogo task, and secondly to explore whether there was any evidence that the two component processes develop differently by collecting data from 8-year-olds and adults. While our findings in part concur with and in part deviate from previous findings in this field, overall they support the existence of separable component processes that develop differently. First we summarize the results regarding interference suppression and response inhibition in adults, followed by how these effects appear in children and may change with age, before highlighting the contribution that additionally analyzing the LRP can make to our understanding of these processes.

Based on the (albeit limited) existing evidence in this field, we expected to observe in adults an interference suppression N2 effect that would be reflected by a greater N2 in response to Go congruent trials than Go incongruent trials, and would occur relatively late and be centrally located. We found that the N2 was more negative for Go congruent trials than for Go incongruent trials, in accordance with Brydges’ et al. [6] findings and others [18,19]. However, there was no evidence that the interference suppression N2 effect was late occurring or maximal at central sites. Indeed, if anything, the examination of the topography (Fig 4F) suggests that this effect is more frontally distributed (although the ANOVA did not show any significant effect of mean amplitudes on location). Thus, our findings regarding the interference suppression effect in adults both deviate and concur with those of Brydges et al. [6].

Remaining with the results of the adult group, we did observe the expected response inhibition N2 effect–the N2 was greater in response to Nogo congruent trials than Go congruent trials. However, in contrast to Brydges et al. [6] we found this effect to be maximal at central locations rather than frontal. It appears that the adults in our experiment actually recruited a diffuse network for response inhibition (Fig 4F), involving both frontal and parietal networks. This result could be explained by the superordinate cognitive control network hypothesis [16,17]. Using a meta-analytic approach, Niendam [16] showed that tasks requiring inhibitory control activate a large network of frontal and parietal regions related to cognitive control and suggested that parietal activation is used for processing stimulus-response pairings. It is possible that the different task demands required to identify the relevant stimulus feature (in our experiment, the type of animal, in Brydges et al. the colour of the fish) led to a different network of activation (in our experiment more central, in Brydges more frontal).

There was also no evidence that the response inhibition effect peaked earlier than the interference suppression effect, contrary to the findings of Brydges et al.[6]. Brydges et al. suggest that response inhibition should occur before interference suppression because it requires a complete shut down of all responses (‘do not press any button’) compared to a change in response that should be more complex in the case of interference control. They also suggest that the difference in timing could be due to the type of discrimination used for the cue and stimuli rather than the inhibition effect itself. Brydges et al.’s [6] paradigm used colour discrimination for the stimuli and form discrimination for the cue and they suggest that their finding could be due to the fact that colour processing is faster than form processing. In our task, the cue was defined by location (above or below a fixation point) and the discrimination of the stimuli involved both colour and shape (blue vs. orange animal and animal from the land vs. animal from the sea). Because selection by location is faster than discrimination by colour and shape [43], it is possible that this delayed the timing of when response inhibition was required, and resulted in no difference in latency between response inhibition and interference control using our paradigm. However, other researchers such as Friedman and Miyake [44] theorized that interference suppression should actually occur before response inhibition so it seems that the timing of these effects need further investigation.

Thus, our findings partly concur and partly deviate with those of Brydges et al. [6]. As well as the differences between the two paradigms already mentioned, the age difference between the studies might also partly explain these differences. Because the adult group in Brydges et al. [28] consisted of 18 year olds and because the brain is still maturing at that age with increases in myelination until 21 years and decreases in gray matter in the frontal cortex until at least 23 years [30], it is possible that their adult group was still maturing whereas our adult group was not. The use of a more controlled paradigm and a wider age-range of participants could help address these issues.

When we examined the N2 elicited by this task in children, we observed that children did not show a significant interference suppression N2 effect, but they did show a response inhibition N2 effect qualitatively the same as that observed in adults. Other studies have also reported no interference suppression N2 effect–Krämer and colleagues [5] in an adapted Stop Signal task, and Brydges and colleagues [28] in children using their adapted Go/Nogo flanker task. In the Introduction we speculated that the latter finding may have been due to certain methodological features of the Brydges study, however having improved upon these issues we still failed to find an interference suppression N2 effect in children. Thus, perhaps this finding indicates that interference suppression is a later developing skill than response inhibition. This is consistent with and extends the findings of Brydges et al. [28], who failed to find an interference suppression N2 effect in 11-year-old children. Additional developmental studies including more age groups could further broaden our understanding of the development of interference suppression.

The lack of a significant interference suppression N2 effect could be considered surprising given the strong influence the cue had on response preparation in children (LRP results). That is, in Go incongruent trials, children prepared an incorrect response during the pre-stimulus period, and then had to overcome this response preparation to give the correct response when the stimulus was presented. The fact that this was not accompanied by a significant difference in N2 amplitude is rather unexpected. One interpretation of this result pattern is that due to immature interference suppression skills, children do not process Go incongruent trials in the same way as do adults and recruit parietal rather than frontal regions. Bunge et al. [2] found that adults recruited different brain regions from children, due to a shift in cognitive strategies during this period. Perhaps our child participants were using a range of processes in the Go incongruent condition, depending on their developmental stage, which lead to a rather heterogeneous group and therefore no significant interference suppression N2 effect.

In children, the difference in N2 amplitude between Nogo congruent and Go congruent conditions suggests that by 8 years of age, response inhibition is qualitatively the same as in adults. Nevertheless, a study by Lamm, Zelazo, & Lewis [45] demonstrated that age-related decreases in N2 amplitude reflect the development of cognitive control (and not only skull thickness, for instance) which suggests that response inhibition was probably still under development in our child group, as they showed much larger N2 amplitudes compared to the adult group. We suggest that the reduced N2 amplitude and latency observed in our experiment in adults vs. children reflect reduced cognitive demand [46] and increased myelination [47], respectively. Furthermore, the topography results in the present study could suggest a developmental trend, with the response inhibition effect being maximal at frontal locations in children and at central locations in adults. However, this was not statistically supported by the mean amplitude data.

We also found an interesting developmental trend in our LRP data, which was not originally expected. We initially expected to see a LRP preparation in the direction of the cue for both the children and the adults. However, only the children showed a motor response preparation in the direction of the cue and the adults showed no response preparation in response to the cue in any condition. There is, however, plenty of evidence that the adults were influenced by the cue, as we observed reaction time and N2 amplitude differences between the Go congruent and Go incongruent conditions. This indicates that the influence of the cue simply did not reach the motor stage in adults. It is possible that the mechanism involved was more automatic and could be related to what is known as the Simon effect [48]. This effect is generally observed in choice reaction time tasks and shows that reactions are faster when stimulus and response locations are on the same side (ipsilateral) than when they are contralateral. Valle-Inclán & Redondo [49] showed that the Simon effect could be observed in reaction time without any observation of motor response preparation as indexed by the LRP. Although unexpected, our results suggest an interesting developmental pattern with the children in our study using a proactive strategy, and the adults using a reactive strategy [50]. That is, the adults, although influenced by the cue, waited until they had all the necessary information before they prepared a response, which might explain why they made fewer mistakes than the children.

While the present study contributes to our understanding of inhibitory control and how it develops, it has some weaknesses that deserve acknowledgment. Primarily, the inclusion of only two age groups limited our ability to draw conclusions about developmental change. Collecting data from more age groups using this or similar tasks would allow us to more closely examine both the development of the interference suppression N2 effect and the effect of the cue on response preparation. For now, we can simply conclude that interference suppression is not yet developed in 8 year olds. Additionally, we speculated that the 8 year olds might have been using a range of processes in the Go incongruent condition, depending on their developmental stage. Because of the small sample, we could not investigate this further and future studies wishing to examine this should aim for a larger sample size. Finally, we note that the children’s data was probably noisier than the adults’ data, due to the original difference in the number of trials and age differences in the ability to meet task demands (e.g. sit still) further reducing the number of trials included for ERP analysis. However, by examining mean rather than peak amplitude and by having a high number of trials, we reduced the chance of selecting unintended maxima due to noise. We suggest that future studies investigating the development of inhibitory control include more than two age groups, increase the sample size of each, and use a high number of trials for each conditions.

## Conclusion

Our aim was to evaluate the existence of two component processes of inhibitory control–interference suppression and response inhibition–using a single task measuring both processes. We also wanted to investigate any developmental changes in these two processes. We found a significant interference suppression N2 effect in the adults only, and a response inhibition N2 effect in both children and adults. Further, a cue (which could be congruent or incongruent) influenced the motor stages of processing in children, but not in adults. Our results support the idea that there are two separable component processes of inhibitory control, and that they develop differently, with interference suppression developing later than response inhibition.

## Supporting Information

S1 File ERP mean amplitude and latencies for each condition. MA: mean amplitude, LAT: Latency; FCz: Fronto-central electrode; Fz: Frontal electrode; Cz: Central electrode; GC: Go Congruent; GI: Go Incongruent; NGC: Nogo Congruent; NGI: Nogo Incongruent.
(XLSX)

S2 File Reaction time and Accuracy for each condition. RT: Reaction Time; GC: Go Congruent; GI: Go Incongruent; NGC: Nogo Congruent; NGI: Nogo Incongruent.
(XLSX)
